# Simultaneous Analysis of SEPT9 Promoter Methylation Status, Micronuclei Frequency, and Folate-Related Gene Polymorphisms: The Potential for a Novel Blood-Based Colorectal Cancer Biomarker

**DOI:** 10.3390/ijms161226113

**Published:** 2015-12-01

**Authors:** Gloria Ravegnini, Juan Manuel Zolezzi Moraga, Francesca Maffei, Muriel Musti, Corrado Zenesini, Vittorio Simeon, Giulia Sammarini, Davide Festi, Patrizia Hrelia, Sabrina Angelini

**Affiliations:** 1 Department of Pharmacy and Biotechnology, University of Bologna, via Irnerio 48, 40126 Bologna, Italy; gloria.ravegnini2@unibo.it (G.R.); juan.zolezzimoraga@gmail.com (J.M.Z.M.); giulia.sammarini2@unibo.it (G.S.); patrizia.hrelia@unibo.it (P.H.); 2 Laboratorio de Biología Celular y Molecular, Departamento de Biología, Facultad de Ciencias, Universidad de Tarapacá, Arica 1000007, Chile; 3 Department for Life Quality Studies, University of Bologna, Corso d’Augusto 237, 47921 Rimini, Italy; francesca.maffei@unibo.it; 4 Unit of Epidemiology, Health Promotion and Risk Communication, Department of Public Health, Bologna Local Health Authority, Via del Seminario1, 40068 San lazzaro di Savena, Italy; m.musti@ausl.bologna.it; 5 Unit of Epidemiology and Biostatistics, IRCCS, ISNB, Via Altura 3, 40139 Bologna, Italy; zenesinicorrado@gmail.com; 6 Laboratory of Pre-Clinical and Translational Research Reference Cancer Center of Basilicata, Scientific Institute of Hospitalization and Treatment, Rionero in Vulture, 85028 Potenza, Italy; vittorio.simeon@crob.it; 7 Department of Clinical Medicine, University of Bologna, Via Massarenti 9, 40138 Bologna, Italy; davide.festi@unibo.it

**Keywords:** SEPT9 methylation, micronuclei, genetic polymorphisms, colorectal cancer

## Abstract

One challenge in colorectal cancer (CRC) is identifying novel biomarkers to be introduced in screening programs. The present study investigated the promoter methylation status of the SEPT9 gene in peripheral blood samples of subjects’ positive fecal occult blood test (FOBT). In order to add new insights, we investigated the association between SEPT9 promoter methylation and micronuclei frequency, and polymorphisms in the folate-related pathway genes. SEPT9 promoter methylation, micronuclei frequency, and genotypes were evaluated on 74 individuals’ FOBT positive. Individuals were subjected to a colonoscopy that provided written informed consent for study participation. SEPT9 promoter methylation status was significantly lower in the CRC group than controls (*p* = 0.0006). In contrast, the CaCo2 cell-line, analyzed as a tissue specific model of colon adenocarcinoma, showed a significantly higher percentage of SEPT9 promoter methylation compared to the CRC group (*p* < 0.0001). Linear regression analysis showed an inverse correlation between micronuclei frequency and the decrease in the methylation levels of SEPT9 promoter region among CRC patients (β = −0.926, *p* = 0.0001). With regard to genotype analysis, we showed the involvement of the DHFR polymorphism (rs70991108) in SEPT9 promoter methylation level in CRC patients only. In particular, the presence of at least one 19 bp del allele significantly correlates with decreased SEPT9 promoter methylation, compared to the 19 bp ins/ins genotype (*p* = 0.007). While remaining aware of the strengths and limitations of the study, this represents the first evidence of a novel approach for the early detection of CRC, using SEPT9 promoter methylation, micronuclei frequency and genotypes, with the potential to improve CRC risk assessment.

## 1. Introduction

The pathogenesis of colorectal cancer (CRC) is very complex, with many different pathways actively involved in the carcinogenic process. Genetic instability plays a pivotal role in these different pathways, with chromosomal instability, microsatellite instability, and abnormal DNA methylation as the mayor path described for CRC [1]. The diagnosis of CRC at early stages is one of the proven strategies resulting in a higher cure rate [2]. However, although CRC can be prevented by screening programs, only 39% of cases are diagnosed at early stages, highlighting the urgent need for new biomarkers of early CRC detection [3]. Current adopted CRC screening procedures include the fecal occult blood test (FOBT) and colonoscopy. The sensitivity and specificity of FOBT is not as good as colonoscopy, which has the highest efficacy, but is invasive, and its acceptance by the general population is rather low. Therefore, in an attempt to improve actual screening procedures, some studies have identified potential novel cancer markers in serum, plasma, and stool [4,5,6]. Micronucleus (MN) frequency in peripheral blood lymphocytes (PBL) has emerged as one of the most reliable biomarkers for cancer risk assessment, including CRC [7]. Besides genetic events, epigenetic changes, including DNA methylation, histone modifications, and deregulation of non-coding RNAs may play a crucial role in tumor progression [8,9]. In recent years, DNA methylation and its role in tumorigenesis has emerged as a hallmark in molecular oncology, including global genomic hypomethylation and CpG-promoter hypermethylation of tumor suppressor genes [10]. DNA methylation-based tests appear to have a promising role in early CRC detection to be used in screening programs [11,12]. One of the latest DNA methylation-based biomarkers for CRC is the plasmatic septin 9 (SEPT9) promoter hypermethylation analyses, which has consistently demonstrated utility in detecting CRC in several clinical studies [13,14]. Although the regulation of gene expression by aberrant methylation has been extensively described in CRC, further studies are necessary to validate the use of methylation biomarkers for the early detection of CRC. In this regard, we investigated the promoter methylation status of the SEPT9 gene in peripheral blood samples of subjects who tested positive in the FOBT test, and were subsequently clinically classified by colonoscopy examination. In addition, in order to improve our knowledge and add new insights in the development of new biomarkers related to CRC risk, we also investigated the association between SEPT9 promoter methylation status and MN frequency in PBL. Furthermore, as the origin of altered methylation remains largely unknown, we tested the genetic hypothesis of an influence of polymorphisms in the folate-related genes, on SEPT9 promoter methylation status.

## 2. Results and Discussion

**Figure 1:**
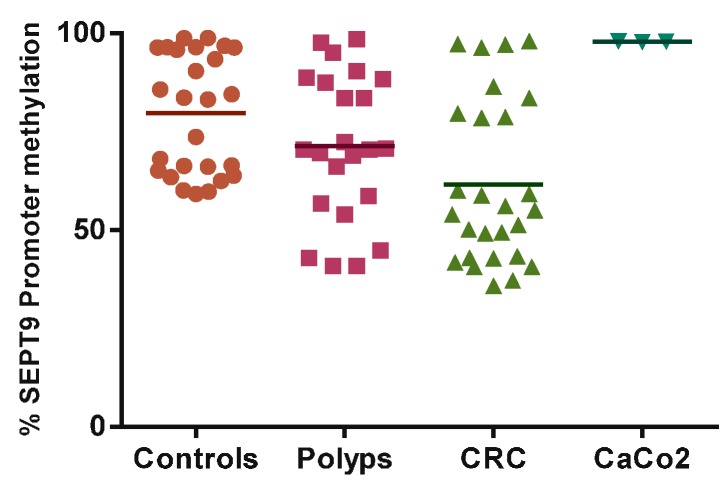
SEPT9 promoter methylation status in controls, polyps, colorectal cancer (CRC) and CaCo2 cell line.

SEPT9 promoter methylation status was significantly lower in peripheral blood DNA of the CRC group than controls (61.64% ± 20.88% *vs.* 79.71% ± 15.15%, *p* = 0.0006; Figure 1).

**Figure 2:**
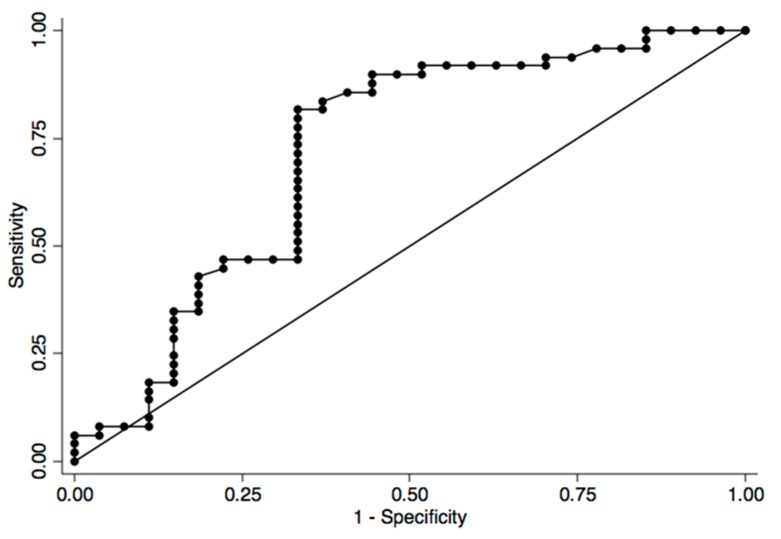
ROC curve was applied to test specificity and sensitivity.

In contrast, the CaCo2 cell-line, analyzed as a tissue specific model of colon adenocarcinoma, showed a significantly higher percentage of methylation status in the SEPT9 promoter region compared to the CRC group (97.95% ± 0.06% *vs.* 61.64% ± 20.88%, *p* < 0.0001; Figure 1). We further examined the potential of this promising marker by constructing the ROC curve. The area under the curve was 0.77 (95% CI 0.64–0.91) and the optimal cut-off of the SEPT9 promoter methylation was 60%, reaching a specificity of 66.7% and achieved a sensitivity of 92.3%. We further examined diagnostic potentials of this promising marker, using a logistic regression model. The SEPT9 promoter methylation (<60%) was discriminative between CRC patients and healthy controls (OR 20.4, 95% CI 3.96–105.21, *p* < 0.001; Figure 2).

**Figure 3:**
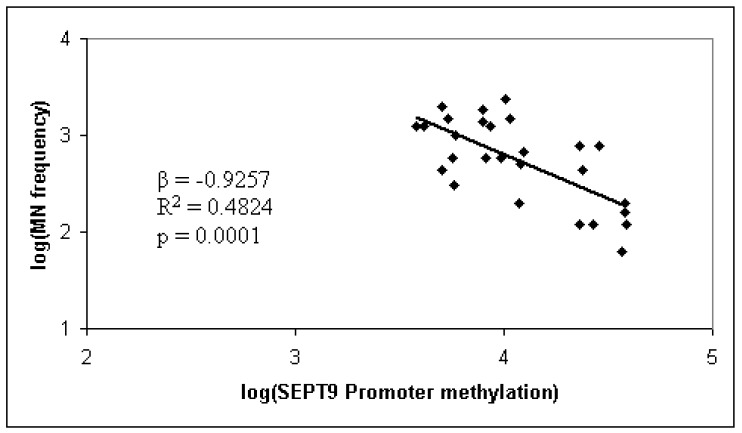
Linear regression analysis showed an inverse correlation between micronucleus (MN) frequency and the decrease in methylation levels of SEPT9 promoter region among colorectal cancer (CRC) patients.

SEPT9 has very unique characteristics; in fact literature supports its activity as an oncogene, although more recently tumor suppressor functions have been reported [15]. In particular, SEPT9 overexpression has been reported in different human tumors, including breast, ovary, and blood; on the other hand, SEPT9 silencing, through a mechanism of promoter hypermethylation, has been reported in colorectal and head and neck cancer [16,17,18,19]. In the present study, cancer-tissue specific SEPT9 hypermethylation in CRC is confirmed by the finding on the CaCo2 cell-line. With regard to SEPT9 methylation status, we found hypermethylation in peripheral blood DNA of the control group, and a lower methylation in CRC patients. Indeed, there is a growing literature showing the loss of global methylation associated with hypermethylation of the promoter of specific genes occurring in carcinogenesis [20]. Furthermore, case-control studies have highlighted a lower global DNA methylation in cancer patients compared to the controls. Accordingly, we may assume a SEPT9 cancer-tissue specific (*i.e.*, CaCo2 cells, or DNA from plasma) hypermethylation and, at the same time, its hypomethylation in peripheral blood DNA. These results take on an even greater significance given that the percentage of methylation in the colorectal polyps group ranks in between the group of control and CRC. This finding led us to consider the possibility that SEPT9 methylation, in peripheral blood DNA, follows the different main stages of the pathology—healthy => adenoma => CRC, as we have previously found for MN frequency [21]. The opportunity to use peripheral blood samples is unique. In fact, through its use as surrogate tissue, which requires non-invasive procedures normally well accepted by the general population, large-scale screening studies could become feasible. SEPT9 promoter hypermethylation analysis represents one of the most promising DNA methylation-based plasmatic biomarker for CRC. However, despite the different positive results reported in the literature [13,16,17,19] and some commercially available tests, there is still concern about their application in the general screening settings. In particular, the main issue relates to the sensitivity as, for example, detection rate of early stage CRC and large adenomas by a multi-marker stool DNA test, which included methylated BMP3, NDRG4, and TFPI2, appear to be higher than rates by plasmatic methylated SEPT9 test [18]. In an attempt to overcome this issue, for the first time we analyzed the correlation between MN frequency in PBL and SEPT9 promoter methylation status. Previous studies have highlighted the existing relationship between MN increase frequency and histopathological changes in CRC carcinogenesis, supporting the use of MN frequency as a biomarker for CRC risk-assessment [21,22]. Although MN frequency shows the potential to recognize individuals at a higher risk of developing cancer, so that they may benefit from early detection and prevention programs, its translation into the practice still has not occurred. With regards to CRC, considering that it clearly develops through a multistep process involving the progressive accumulation of genetic and epigenetic alterations, we thought to refine the CRC risk assessment adding methylation profile. In the present study, linear regression analysis showed an inverse correlation between the MN frequency and the decrease in methylation levels of SEPT9 promoter region among CRC patients (β = −0.926, *p* = 0.0001; Figure 3).

A similar relationship, but at a lower level of significance was observed in patients with colorectal polyps (β = −0.478, *p* = 0.022). No correlation was observed between MN frequency and SEPT9 promoter methylation in controls (data not shown). To the best of our knowledge this represents the first study investigating simultaneously MN frequency and the methylation percentage in the promoter region of the SEPT9 gene, in peripheral blood samples from CRC patients, colorectal polyps individuals, and controls.

The concerted action of enzymes that control folate uptake and metabolism are essential for DNA methylation, synthesis, and repair. Folate-related genes are characterized by a high degree of genetic variability, which in part might explain inter-individual variability in folate uptake and metabolism. Few studies have associated polymorphisms in these genes with DNA strand breaks, chromosomal damage, and micronucleus formation in lymphocytes [23,24,25]; in addition, they have been associated with cancer susceptibility, including colorectal and breast cancer, gastrointestinal stromal tumor and cancers of the esophagus, stomach, and liver [26,27,28,29,30]. From this perspective, we analyzed whether MN frequency and SEPT9 methylation in CRC patients was associated with genetic variants in relevant genes belonging to the folate pathway. Our results did not show any significant association between MN frequency and genetic polymorphisms (data not shown); however, while is well known that low folate status is associated with DNA damage, whether these effects may also depend on variants in folate uptake and metabolism genes remains largely uncertain. With regard to polymorphisms analysis, genotype frequencies were found to be in Hardy-Weinberg equilibrium (HWe) in both CRC patients and controls. Minor allele frequencies (MAF) and HWe *p*-value are presented in Table S2. The observed genotype frequencies are consistent with the one reported in the public HapMap—CEU population recorded on Pubmed (http://www.ncbi.nlm.nih.gov/snp), showing that the MAF herein reported are representative and not due population selection. Details of the results of association tests for each genetic polymorphisms are presented in Table 1.

**Table 1:** Genotype distribution of the folate-related polymorphisms in controls and colorectal cancer (CRC) patients.

Genotype	CRC *n* (%)	Controls *n* (%)	OR (95% CI)	*p*
**RFC rs1051266, p.His27Arg**				
AA	8 (33.3)	9 (34.6)	1.00	1.000
AG	12 (50.0)	13 (50.0)	1.04 (0.26–4.25)	
GG	4 (16.7)	4 (15.4)	1.11 (0.15–8.32)	
AG/GG	16 (66.7)	17 (65.4)	1.06 (0.28–4.03)	
**FOLR rs20710110, −20G>A**				
AA	23 (95.8)	22 (84.6)	1.00	0.351
AG	1 (4.2)	4 (15.4)	0.25 (0.01–2.74)	
**DHFR rs70991108, 86+59_86+60insACCTGGGCGGGACGCGCCA**				
19+/+	13 (54.2)	7 (26.9)	1.00	0.144
19+/−	9 (37.5)	14 (53.9)	0.36 (0.08–1.41)	
19-/−	2 (8.3)	5 (19.2)	0.23 (0.02–1.85)	
19+/−_19−/−	11 (45.8)	19 (73.1)	0.32 (0.08–1.17)	
TS 28BP rs45445694. *34+169_*34+196CGGGACGGAGGCAGGCCAAGTGGCGCGG [2,3]				
2R2R	6 (25.0)	4 (15.4)	1.00	0.013
2R3R	16 (66.7)	10 (38.5)	1.06 (0.17–5.90)	
3R3R	2 (8.3)	12 (46.1)	0.12 (0.01–1.05)	
2R3R/3R3R	18 (75.0)	22 (84.6)	0.55 (0.10–2.75)	
**SHMT rs1979277, p.Leu474Phe**				
CC	16 (66.7)	14 (53.9)	1.00	0.686
CT	7 (29.2)	11 (42.3)	0.56 (0.14–2.12)	
TT	1 (4.1)	1 (3.9)	0.88 (0.01–73.53)	
CT/TT	8 (33.3)	12 (46.2)	0.59 (0.16–2.11)	
**MTHFR rs1801133, p.Ala222Val**				
CC	7 (29.2)	6 (23.1)	1.00	0.867
CT	13 (54.2)	14 (53.8)	0.80 (0.17–3.65)	
TT	4 (16.6)	6 (23.1)	0.59 (0.08–4.00)	
CT/TT	17 (79.8)	20 (76.9)	0.73 (0.17–3.11)	
**MTHFR r1801131, p.Glu429Ala**				
AA	15 (62.5)	11 (42.3)	1.00	0.424
AC	8 (33.3)	13 (50.0)	0.46 (0.12–1.69)	
CC	1 (4.2)	2 (7.7)	0.38 (0.01–8.16)	
AC/CC	9 (37.5)	15 (57.7)	0.45 (0.12–1.57)	
**MTRR rs1801394, p.Ile49Met**				
AA	13 (54.2)	6 (23.1)	1.00	0.032
AG	9 (37.5)	12 (46.1)	0.36 (0.08–1.50)	
GG	2 (8.3)	8 (30.8)	0.13 (0.01–0.89)	
AG/GG	11 (45.8)	20 (76.9)	0.26 (0.06–0.99)	

The MTRR variant allele (rs1801394-GG) and the TS polymorphism (rs45445694-3R3R) were associated with a decreased susceptibility to CRC (OR 0.13, 95% CI 0.01–0.89; *p* = 0.032 and OR 0.12, 95% CI 0.01–1.05; *p* = 0.013, respectively) compared to the wild-type genotype (AA and 2R2R). We are aware that the small sample size has made it impractical to make multiple adjustments and might have increased the probability of detecting an association by chance, however, both the polymorphisms have functional significance, and therefore their role in cancer risk is plausible. In particular, the well-studied MTRR polymorphism, changing isoleucine to methionine, is associated with risk of most widespread cancer types, including gastric, colon and prostate cancer, and leukemia [31,32,33,34]. With regard to the TS polymorphisms, *in vitro* studies associated the 3R allele with a higher TS expression. Deficiencies in the TS enzyme increase the rate of misincorporation of uridylate into DNA, causing DNA strand breaks and other chromosomal damage, thus increasing the predisposition to cancer. Notably, this is in agreement with our finding and other studies reported in the literature on CRC [35,36,37], however the limited sample size does not allow for making any definitive conclusions, therefore this should be considered as an explorative study and the finding deserves further investigation.

**Figure 4:**
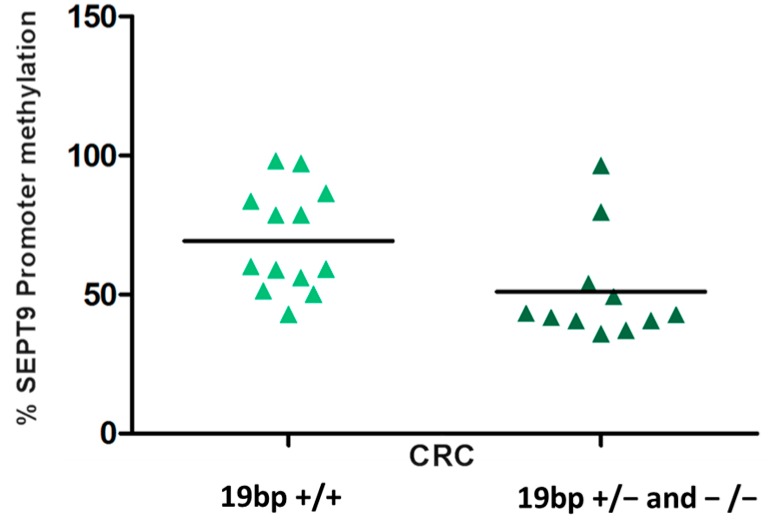
Presence of the 19 bp del allele (19 bp ins/del plus 19 bp del/del) is significantly correlated with decreased SEPT9 promoter methylation compared to the 19 bp ins/ins genotype.

Given the critical role in the DNA methylation process, the folate pathway has been largely investigated as a potential modulator of DNA methylation [29,38]. In particular, several studies have assessed the influence of genetic polymorphisms in folate-related genes as modulators of DNA methylation changes in key *CRC* genes [37,39,40,41]. A very interesting finding of the present study is the involvement of the DHFR polymorphism (rs70991108) in the SEPT9 promoter methylation level in CRC patients only. Particularly, the presence of the 19 bp del allele (19 bp ins/del plus 19 bp del/del) significantly correlates with decreased SEPT9 promoter methylation compared to the 19 bp ins/ins genotype (51.06% ± 19.33% *vs.* 69.31% ± 18.55%; *p* = 0.007; Figure 4).

None of the other selected polymorphisms influenced the SEPT9 methylation status. The DHFR enzyme catalyzes the reduction of dietary folic acid or dihydrofolate to tetrahydrofolate (THF), the predominant form of folate in plasma required for nucleic acid synthesis and methylation reaction. Thus, it is plausible that DHFR polymorphisms might affect methylation levels also dependent on folate status. Presently *in vitro* analyses of the DHFR variant enzyme activity are lacking; however, we might speculate this result could represent a link between nutritional factors, epigenetic, genetics and cancer development.

In summary, our data highlight that combining SEPT9 promoter methylation in peripheral blood DNA with MN frequency has the potential to be a sensitive and non-invasive biomarker for CRC risk assessment. With regard to polymorphisms analysis in folate related genes, currently, there are controversial data available in the literature pertaining to their possible role in cancer risk, and hardly any tangible data on CRC risk, folate intake and genetic polymorphisms. Our finding should be considered in the context of both strengths: investigation of a comprehensive number of polymorphisms across a logical sequence of genes with well-known roles in the folate pathway—and limitations—the small sample size, and should therefore viewed as exploratory. Aware of the limitation of the study, nevertheless we are convinced that data presented here have to be considered as a starting point and should encourage further investigation.

In addition, considering the bias associated with MSP analysis [42], used in our sample set, further SEPT9 epigenetic studies on a large population size, applying unbiased analyses, as the “methylation-insensitive primers” (MIPs), are warranted.

The SEPT9 sequence we analyzed included several TF sites (retrieved from http://alggen.lsi.upc.es/), which may be physically impeded by DNA methylation and, consequently, gene transcription may inhibit results. In any case, even in the absence of TF site, Methyl-CpG binding domain proteins may bind methylated DNA, causing the recruitment of additional proteins into the locus forming compact, inactive chromatin, resulting in in transcription inhibition [43,44]. In view of this consideration, future studies should also consider the analysis, on the same sample of SEPT9 DNA methylation and expression in order to strengthen any finding.

In conclusion, our findings confirmed that SEPT9 promoter methylation and MN frequency, both measured in peripheral blood, occur at an early stage compared to carcinoma development, indicating that the approach might be suitable to monitor CRC development, and to refine the management of CRC patients We also observed an interesting correlation between markers of the folate pathway and SEPT9 promoter methylation in CRC, further supporting the hypothesis of genetic and epigenetic interaction.

## 3. Methods

### 3.1. Study Population

Comprehensive demographic, dietary, and lifestyle data were collected by physician interviewers through the use of a questionnaire. From an original sample set of 82 subjects, we were able to evaluate 74 individuals. As previously reported [21], the study population comprises three groups of individuals selected among the voluntary participants, aged 50–70 years, of a CRC screening program organized by a specialized physician from the Department of Clinical Medicine at the Sant’Orsola Malpighi Hospital, University of Bologna. Selected demographic characteristics of the study population are reported in Table 1. In particular, the 74 individuals, that first resulted positive to FOBT and, consequently, have been subjected to colonoscopy, were stratified according to histological lesion at the baseline colonoscopy in three groups: (*i*) controls, with normal epithelium (*n* = 26, 7 females and 19 males); (*ii*) patients with colorectal polyps (*n* = 23, 10 females and 13 males); and (*iii*) CRC patients (*n* = 27 subjects, 10 females and 17 males) (Table 2).

Peripheral blood samples were collected from each subject before colonoscopy, and transferred to the laboratory within few hours for subsequent methylation analysis and micronucleus assay. All participants were part of a CRC screening program, started in the Emilia-Romagna region in 2005, and directed to resident and domiciled of both sexes aged 50–70. The target population was invited by regular letter to carry out the FOBT [45]. This research project was conducted only on those individuals FOBT positive and subjected to colonoscopy that provided written informed consent for study participation and anonymous data publication in accordance with national legislation. Any subjects could cancel participation at any time during the study, according to Helsinki Declaration and later Amendments.

**Table 2:** Demographic characteristics of the study population.

Characteristics		Controls	Colorectal Polyps	CRC
**Subjects**		26	23	27
**Age**	Mean ± SD	59.62 ± 5.82	60.43 ± 6.14	67.52 ± 9.61
	Range	51−70	48−70	53−88
**Gender *n* (%)**	Female	7 (26.9%)	10 (43.5%)	10 (38.5%)
	Male	19 (73.1%)	13 (56.5%)	16 (61.5%)
**BMI**	Mean ± SD	25.65 ± 3.31	26.88 ± 3.09	24.51 ± 3.48
	Range	18.37−32.42	22.03−35.16	19.61−33.73
**Smoke *n* (%)**	No	19 (73.1%)	18 (78.3%)	18 (69.2%)
	Yes	7 (26.9%)	5 (21.7%)	8 (30.8%)

### 3.2. Methylation Analysis

The analysis was performed on genomic DNA extracted from fresh peripheral blood (FOBT positive individuals) and CaCo2 cells, included as a colorectal cancer cell model, using ZR Genomic DNA (Zymo Research, Irvine, CA, USA). Bisulfite conversion, (approximately 500 ng DNA) was achieved using the EZ DNA Methylation Kit (Zymo Research) following manufacturer’s instructions. If not used immediately, converted DNA was stored at −20 °C for up to one week. Methylation analysis was performed by relative quantitative PCR, in particular using Real-time Methylation Specific-PCR (MSP) primers in a 7900HT Real-Time PCR System (Life Technologies, Carlsbad, CA, USA). Amplified sequences were located within the promoter region of SEPT9 gene, containing 14 CpG sites, and in a non-CpG-containing region of myoD gene, taken as control gene. PCR amplification was carried out in triplicate for each sample. PCR total volume was 25 µL, containing 1 µL bisulphite-converted DNA (10 ng total DNA), 12.5 µL 2X Maxima SYBR green/ROX qPCR master mix (Thermo Fisher, Waltham, MA, USA), SEPT9 primers (forward and reverse; final concentration 0.3 µM), and DNAase-free water. myoD and methylated (M) and unmethylated (U) SEPT9 specific primer sequences were as follows: SEPT9 MFwd-TTAAGTTTAAGGAAATCGTAGTATCG and MRev-AACCACCGAATCTACCTACGAA, (product size 263 bp); UFwd-AATTTTTAAGTTTAAGGAAATTGTAGTATT and URev-CAAACCACCAAATCTACCTACAAA (product size 254 bp); myoD Fwd-TGATTAATTTAGATTGGGTTTAGAGAAGGA, Rev-CCAACTCCAAATCCCCTCTCTAT (product size 162 bp). SEPT9 sequences were retrieved from the online public database http://www.ensembl.org and the web-based software MethPrimer (http://itsa.ucsf.edu/urolabMethPrimer) was used to retrieve primers. Additionally, qRT-PCR was performed using the following cycling conditions: activation at 95 °C for 10 min, 40 cycles of 95 °C for 15 s, 50–65 °C for 30 s, and a final 72 °C for 60 s. Amplification products were verified by melting curve analysis and the correct length and purity of PCR products were verified by agarose-gel electrophoresis. For relative quantification, standard curves were generated separately for each gene using universally methylated/unmethylated DNA (Qiagen, Hilden, Germany). SEPT9 methylation percentage was calculated according to Lehmann and Kreipe [46]. In particular, the percentage of methylation was calculated by the 2^−ΔΔ*C*t^ method, where ΔΔ*C*_t_ = (*C*_t target_ − *C*_t myod_) sample − (*C*_t target_ − *C*_t myod_) fully methylated DNA, multiplying by 100.

### 3.3. Micronuclei Analysis

The MN assay was performed using the cytokinesis-block technique according to well-standardized procedures in our laboratories [21,47]. Two separate cultures from each sample were set up and at least 2000 binucleated (BN) lymphocytes per series of cultures were examined for the presence of one, two or more MN. Data are presented as the frequency of micronucleated BN cells per thousand BNs.

### 3.4. Cell Culture

CaCo2 cells were purchased from IZSLER (Brescia, Italy). The cell line was cultured in Dulbecco’s Modified Eagle Medium (Lonza, Basel, Switzerland) supplemented with 20% heat-inactivated fetal calf serum (Lonza), 1 mM l-glutamine (Sigma Aldrich, St. Louis, MO, USA), 100 UI penicillin and 100 μg/mL streptomycin (Sigma). Cells were grown at 37 °C in humidified atmosphere and 5% CO_2_, until a confluent monolayers was obtained. Cells were trypsinized 0.25% (Trypsin-EDTA solution, Sigma) and aliquoted up to 5 × 10^6^ for DNA extraction and subsequent methylation analysis, as described in the specific paragraphs.

### 3.5. Genotyping Analysis

DNA was extracted from fresh or frozen whole blood using a DNA isolation kit from Qiagen (QIAamp^®^ DNA Mini Kit, Qiagen, Hilden, Germany). Characteristics of the studied polymorphisms—two insertion/deletion, one tandem repeat and ten single nucleotide polymorphisms—are reported in Table S1. Genotypes were determined by polymerase chain reaction (PCR)-based assays (restriction fragment length polymorphism (RFLP) and/or real-time) according to published methods [48,49,50] or as recommended by manufacturer. Positive and negative controls were included in each reaction as quality control. In addition, for internal quality control (accuracy of genotyping), 90% of samples were repeated. The concordance between the original and the duplicate samples for all the analyzed polymorphisms was 100%.

### 3.6. Statistical Analysis

All statistical calculations were performed using Stata Intercooled version 12.0 [51]. The SEPT9 promoter methylation percentage is expressed a mean ± standard deviation. The Kruskal-Wallis test was used to test differences in the SEPT9 promoter methylation percentage between CRC patients, patients with colorectal polyps, and controls. This non-parametric test allows problems related to sample size or normality assumption to be obviated. The Receiver Operating Characteristic analysis (ROC curve) was performed, to estimate the optimal cut-off with sensitivity and specificity, of SEPT9 methylation with the area under the ROC curve being estimated non-parametrically. Linear regression analysis was applied to assess the correlation between the SEPT9 promoter methylation percentage and MN frequency in CRC patients. Regression analysis was performed after logarithmic transformation of non-normally distributed variables. The distribution of genotypes was tested for departures from Hardy-Weinberg equilibrium using χ^2^-test. The Wilcoxon rank-sum test was used to test significant associations between MN and the various genotypes. The frequency distributions of categorical variables were compared using Fisher’s exact test. Odds ratio (OR) with the corresponding 95% confidence interval (95% CI) for assessment of the association between risk and genotype was based on exact logistic regression (adjusting for gender and age). Given the limited sample size of the present study, probability values and additional parameter estimates were not adjusted for multiplicity. Therefore, results should be interpreted as exploratory. Additionally, *p* ≤ 0.05 were considered as significant.

## Supplementary Materials

Supplementary materials can be found at http://www.mdpi.com/1422-0067/16/12/26113/s1.
