# Supplementary material for: Simultaneous Analysis of SEPT9 Promoter Methylation Status, Micronuclei Frequency, and Folate-Related Gene Polymorphisms: The Potential for a Novel Blood-Based Colorectal Cancer Biomarker
Source: Int J Mol Sci. 2015 Dec 1;16(12):28486–97. doi: 10.3390/ijms161226113 (PMC4691060; doi:10.3390/ijms161226113)
Supplement: Supplementary file 1 [file ijms-16-26113-s001.pdf]

Supplementary Materials: Simultaneous Analysis of SEPT9 Promoter Methylation Status, Micronuclei Frequency, and Folate-Related Gene Polymorphisms: The Potential for a Novel Blood-Based Colorectal Cancer Biomarker

Gloria Ravegnini, Juan Manuel Zolezzi Moraga, Francesca Maffei, Muriel Musti, Corrado Zenesini, Vittorio Simeon, Giulia Sammarini, Davide Festi, Patrizia Hrelia and Sabrina Angelini

Table S1. SNPs description according to HGVS recommendations [§].

Gene–RefSNP	HGVS Nomenclature	DNA Variant and Protein	Reference
**RFC–rs1051266**	NC_000021.9:g.45537880T>C	c.80A>G [p.(His27Arg)]	RFLP * [48]
**FOLR–rs2071010**	NC_000011.10:g.72189920G>A	c.-20G>A	RT **
**DHFR–rs70991108**	NC_000005.10:g.80654344_80654345insTG GCGCGTCCCGCCCAGGT	c.86+59_86+60insACCTGG GCGGGACGCGCCA	RFLP * [49]
**TS–rs45445694**	NC_000018.10:g.647646_647673CCGCGCC ACTTGGCCTGCCTCCGTCCCG [2–4,7–9]	c.*34+169_*34+196CGGGACGGAGG CAGGCCAAGTGGCGCGG [2–4,7–9]	RFLP * [50]
**SHMT–rs1979277**	NC_000017.11:g.18328782G>A	c.1420C>T [p.(Leu474Phe)]	RT **
**MTHFR–rs1801133**	NC_000001.11:g.11796321G>A	c.665C>T [p.(Ala222Val)]	RT **
**rs1801131**	NC_000001.11:g.11794419T>G	c.1286A>C [p.(Glu429Ala)]	RT **
**MTRR–rs1801394**	NC_000005.10:g.7870860A>G	c.147A>G [p.(Ile49Met)]	RT **

§ HGVS nomenclature version 2.0, searched through Mutalyzer 2.0.beta-32, released on 26 June 2014; * RFLP = PCR-RFLP analysis carried out according to published methods (reference parenthetically); ** RT = Real-Time PCR with TaqMan allelic discrimination assay (Applied Biosystem, Foster City, CA, USA).

Table S2. Allele frequency in CRC patients (cases) and controls.

**Genotypes Distribution *n***					
**Subjetcs**	**WT**	**HE**	**SNP**	**MAF ***	P_{HWE}
**RFC–rs1051266**					
Cases	8	12	4	*q* = 0.417	0.889
Controls	9	13	4	*q* = 0.404	0.845
**FOLR–rs2071010**					
Cases	23	1	0	*q* = 0.021	0.917
Controls	22	4	0	*q* = 0.077	0.671
**DHFR–rs70991108**					
Cases	13	9	2	*q* = 0.271	0.804
Controls	7	14	5	*q* = 0.461	0.671
**TS–rs45445694**					
Cases	6	16	2	*q* = 0.417	0.069
Controls	4	10	12	*q* = 0.654	0.443
**SHMT–rs1979277**					
Cases	16	7	1	*q* = 0.188	0.834
Controls	14	11	1	*q* = 0.250	0.513
**MTHFR–rs1801133**					
Cases	7	13	4	*q* = 0.437	0.622
Controls	6	14	6	*q* = 0.500	0.695
**MTHFR–rs1801131**					
Cases	15	8	1	*q* = 0.208	0.959
Controls	11	13	2	*q* = 0.327	0.488
**MTRR–rs1801394**					
Cases	13	9	2	*q* = 0.271	0.804
Controls	6	12	8	*q* = 0.538	0.716

* MAF: Minor allele frequency.
